# Establishing Drug Effects on Electrocorticographic Activity in a Genetic Absence Epilepsy Model: Advances and Pitfalls

**DOI:** 10.3389/fphar.2020.00395

**Published:** 2020-04-14

**Authors:** Gilles van Luijtelaar, Gerard van Oijen

**Affiliations:** Donders Centre for Cognition, Radboud University, Nijmegen, Netherlands

**Keywords:** WAG/Rij’s rats, antiepiletic drugs, EEG-behavioral relationship, genetic absence models, spike-wave discharges (SWDs)

## Abstract

The genetic rat models such as rats of the WAG/Rij strain and GAERS were developed as models for generalized genetic epilepsy and in particular for childhood absence epilepsy. These animal models were described in the eighties of the previous century and both models have, among others, face, construct and predictive validity. Both models were and are currently used as models to predict the action of antiepileptic medication and other experimental treatments, to elucidate neurobiological mechanisms of spike-wave discharges and epileptogenesis. Although the electroencephalagram (EEG)/electrocorticogram (ECoG) is imperative for establishing absence seizures and to quantify the for absence epilepsy typical spike-wave discharges, monitoring the animals behavior is equally necessary. Here an overview is given regarding the design of drug evaluation studies, which animals to use, classical and new EEG variables, the monitoring and quantification of the behavior of the rats, some pitfalls regarding the interpretation of the data, and some developments in EEG technology.

## Introduction

Predictive validity is the key word in preclinical animal models. This implies that results of putative new treatment in humans, often new drugs, can be predicted correctly based on results obtained in the models. This was illustrated in the GAERS and WAG/Rij models by showing that the classical anti-absence drugs such as ETX and VPA, as well as benzodiazepines, correctly predicted their action in patients with absences. The outcomes of the studies in the models can be considered as correct predictions, since these antiepileptic drugs suppress dose and time dependently, absence seizures, better, the more easily quantifiable EEG hallmarks of absences, spike-wave discharges (SWDs) in the models. A good EEG signal is imperative for the evaluation of drug studies on SWDs. For an overview of some of the most commonly used antiepileptic drugs that were evaluated in these two models see e.g. Depaulis and van Luijtelaar (2006). Since then very few new antiepileptic drugs were introduced in the market, and none new antiepileptic drugs were specific aimed for the treatment of absence epilepsy. The models were also successfully used to predict an aggravation of SWDs in patients, good examples are the GABA-mimetics tiagabine and vigabatrin, and various sodium channel blockers, such as lamotrigine and the classical anticonvulsants phenytoin and carbamazepine. These results as obtained in the genetic models can be considered as correct rejections based on predictions from the models. There is some discussion regarding whether the results as obtained with lamotrigine were correctly predicted in the models, or whether it is a false rejection, implying that it is effective in patients, but not in the genetic models. Lamotrigine has some efficacy in patients although less than VPA and ETX (Glauser et al., 2010), in WAG/Rij rats a decrease in the incidence of SWDs was found as well, but only at a sedative dose of the compound (van Rijn et al., 1994).

Many other drugs and ligands were tested in the models for studying mechanism and processes affecting absence seizures, such as cytokines (van Luijtelaar et al., 2012a; Kovács et al., 2015; Russo et al., 2014), antidepressant and antipsychotic drugs (Russo et al., 2016), different ion channel openers and blockers, NO synthase inhibitors or NO donors (Przewlocka et al., 1996), nucleosides (Kovács et al., 2014), CB1 receptor agonists (van Rijn et al., 2010; Citraro et al., 2013), positive and negative allosteric modulators of mGluR (Celli et al., 2019; Ngomba and van Luijtelaar, 2018), and neuropeptides (for review see e.g. van Luijtelaar and Zobeiri, 2014; Russo et al., 2016). Currently the models are used to predict the effects of new T-type channel blockers (Tringham et al., 2012; Remen et al., 2016), for the study of antiepileptogenesis (Blumenfeld et al., 2008; Sarkisova et al., 2010; van Luijtelaar et al., 2013; Russo et al., 2016; Leo et al., 2019), for the odyssey toward ultimate causes of absence epilepsy in this model, and for experimental treatment techniques for absence epilepsy such as various types of non-invasive and invasive electrical or magnetic stimulation (Godlevsky et al, 2006; van Luijtelaar and Zobeiri, 2014; van Luijtelaar et al., 2017).

### Absence Seizures

**Figure 1:**
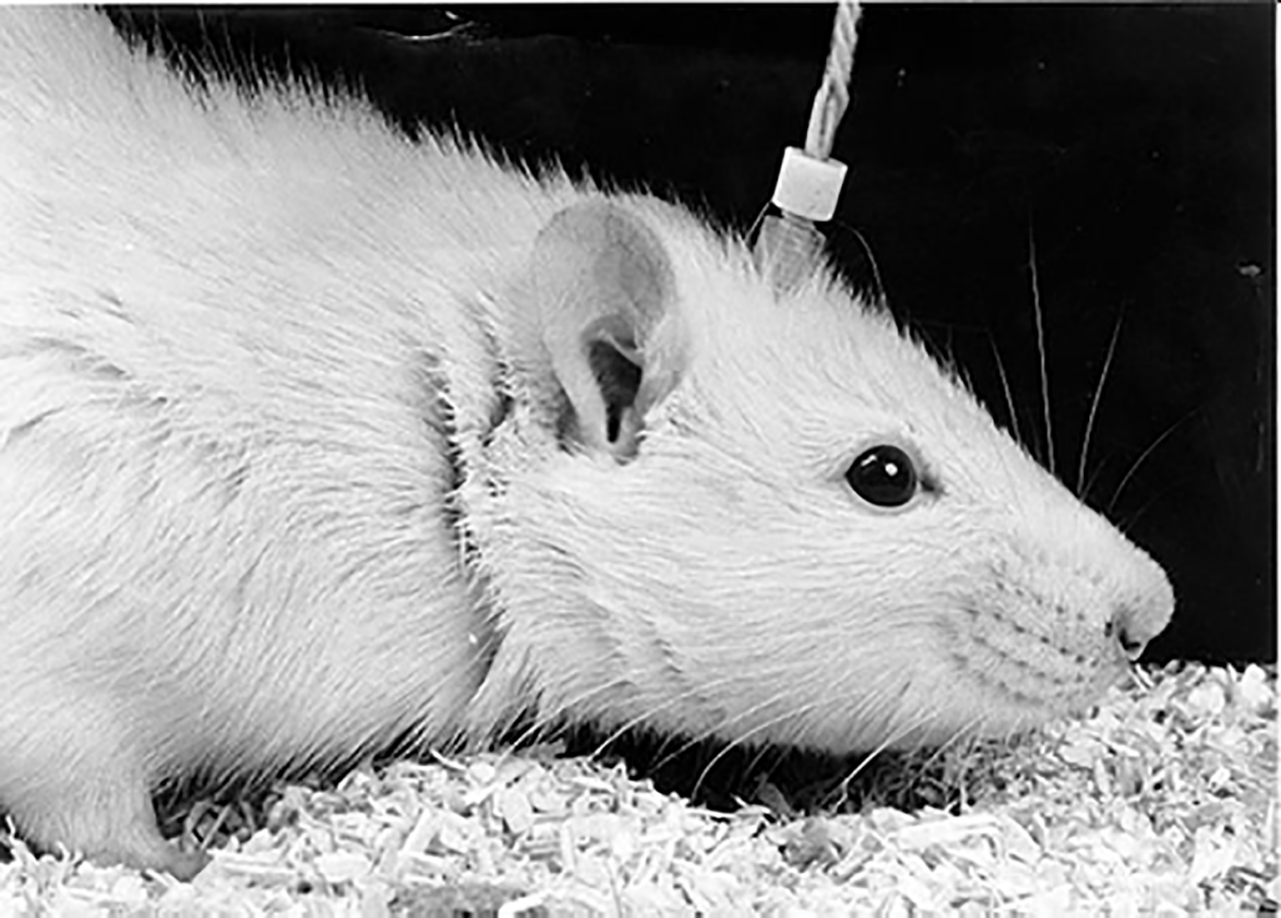
The free moving WAG/Rij rat equipped with a chronically implanted tri-polar Plastic One electrode set connected to a preamplifier and amplifier allowing to quantify number of spike-wave discharges (SWDs) before and after the administration of compounds (Photo H. van de Sluis).

The presence of these absences can only reliably be established with an EEG; absences manifest themselves in the EEG as bilateral synchronized, asymmetric around the zero-line, and generalized SWDs. There are clinical concomitants of SWDs in rodents, but analog to what can be seen in children, these absences remain often unnoticed. Accelerated breathing, rhythmic movements of the vibrissae, incidental eye-blinks, very mild facial myoclonus, and head tilting in an otherwise immobile animal can be noticed concomitant to the SWDs in the EEG in the WAG/Rij model (van Luijtelaar and Coenen, 1986), see **Figure 1**. The trains of SWDs lasting from 1 to up to 30 s, in WAG/Rij the mean duration is about 5 s, sometimes a bit longer (7–8 s, in some labs shorter, ca 3 s) and WAG/Rij’s at 6 months have about 16–20 SWDs per hour, adding several hundred SWDs per 24 h. Shorter than 1 s SWDs are not included by us, since their clinical relevance is not certain, or their morphology remains embryonic. The awareness of elapsing time is disturbed during the SWDs in WAG/Rij rats (van Luijtelaar et al., 1991), suggesting a decrease in the level of consciousness. Here we describe in some detail methods for the evaluation of drugs on the occurrence of the for absence epilepsy typical SWDs with the aid of the EEG, and some pitfalls regarding the interpretation. The most straightforward parameters will be mentioned as well some auxiliary ones, as well as some other approaches regarding data analyses. Finally older and more recent EEG recording techniques in free moving animals will be briefly discussed.

## The Design of The Study: Which Rats to Use

It is crucial that the rats used for the evaluation of a compound have a sufficient amount of SWDs, allowing to establish an increase, and a decrease in seizure activity. In case rats have a low number per time unit, than it might be difficult to further decrease their number. This was the case after rats were chronically exposed to ETX in order to induce antiepileptogenesis. In that case the drug VU0360372 showed only a rather small decrease compared to the action of the same drug in the untreated sham control group (D’Amore et al., 2016). Considering that 2 months old WAG/Rij have few SWDs, and not all animals show clear and unambiguous SWDs, and the age dependent increase in incidence of SWDs, it is recommended to use WAG/Rij’s at an age of 6 months; than it is our experience that they have a sufficient amount of SWD’s to find drug and dose related effects and both an increase and decrease in number and mean duration of SWDs can be found (van Luijtelaar and Coenen, 1986; Schridde and van Luijtelaar, 2004; Bouwman et al., 2007; Remen et al., 2016). In case strong anti-absence effects are anticipated, slightly younger, so 5 months old animals could be used as well. It is not known whether younger animals could be used to establish a pro-absence action. It is our experience though that well known proabsence drugs do not show their pro-absence action in presymptomatic (ca. 2 months old) WAG/Rij rats. GAERS have more SWDs, and they occur more early during ontogeny compared to WAG/Rij rats (Jarre et al., 2017). This has the practical consequence that the base-line (=pre-drug) EEG recording period might be shorter (often 20 min periods were used), and that younger GAERS were successfully used for the evaluation of putative antiepileptic medication. A note of warning is that the incidence of SWDs may vary between labs as a consequence of long term housing in another environment, and genetic drift. Mainly early environmental factors were described affecting incidence and duration in WAG/Rij rats, e.g. maternal (Peeters et al., 1992b; Sitnikova et al., 2016; Sarkisova and Gabova, 2018) and housing effects (Schridde and van Luijtelaar, 2004). Moreover, large differences in incidence were described in the GAERS model between labs in different continents as well (Powell et al., 2014).

### Male Versus Female Rats

The incidence in children with absence epilepsy is larger in girls than in boys, this suggests for a role of the sex chromosomes. Female WAG/Rij rats have as much SWDs per hour as male rats (Coenen and van Luijtelaar, 1987), the same results were obtained in the GAERS model (van Luijtelaar et al., 2014). All the genes involved in absence epilepsy are not discovered as yet and absence epilepsy has a polygenetic origin, none of the known genes are localized at the sex chromosomes. This fact does not preclude the use of female rats. The sex ratio in humans point toward the a sex linked genetic contribution in humans, which is, as mentioned, not present in both Wistar derived models. Female rats and mice are five times less often studied within the field of pharmacology (Beery and Zucker, 2011). Most often indeed male rats of the WAG/Rij and GAERS strain were used to establish effects of drugs; a small disadvantage of the usage of female rats is that the physiological fluctuations in the concentration in the ovarian hormone progesterone in the reproductive 4–5 days cycle modulate the number of spontaneously occurring SWDs in WAG/Rij rats (van Luijtelaar et al., 2001). For about 6 h at proestrus day the number of SWDs is increased compared to the same hours of the other days of the cycle. Specifically at these hours the plasma level of progesterone is enhanced. To control for the ovarian cycle is rather laborious. The higher number of SWDs in some hours of the proestrus days may contribute to a higher between subject variability in acute studies and a higher within subject variability in chronic studies. However, whether this intrinsically more variable number of SWDs in female rats is a sufficient reason for not using females for the evaluation of putative new anti-absence drugs is doubtful since the desired effects of a putative new drug should be robust and a small increase in within and between subjects variability should hardly matter the conclusions whether the new compound is effective or not. Therefore, although female GAERS and WAG/Rij rats were rarely used for drug studies, there are no apriori reasons to use only male subjects. The usage of female subjects for pharmacological studies will also contribute to reducing the number of surplus animals.

Should independent groups of rats be used? Although it is rather common to use independent groups of animals for different doses of a systemically administered compound in an acute pharmacological study, this is not always absolutely necessary. Once rats are implanted with EEG recording electrodes, they can be used repeatedly, among others for different doses of a compound. If this is indeed done, one should control for order effects in order to avoid that order of the drug administration is mixed with dose of the drug. A cross-over block design or counterbalancing for order and dose might prevent this. Next, a sufficient large enough washout period should be used before the animals can be used for a second dose, depending on the half-life of the compound. It is not uncommon to use a 48 h period for this. By starting every new dose with a base-line (=pre-drug) recording session, the putative cross-over, neuroplastic, and wash out effects can be noticed and be controlled for.

It is our experience that a group size of eight animals per dose is generally sufficient to establish reliably the effects of a (dose of a) compound. A solvent control in studying the effects of a compound is very necessary for two reasons: handling animals for the injection, as well as restraining them to connect them with the EEG recording leads, or putting them in a clean EEG recording cage may induce some stress for the animals (especially when mice are used) and stress affects the level of corticosteroids, also in epileptic rats (Tolmacheva et al., 2012). Both stress and steroid hormones are known to have a biphasic effect on SWDs and even the anticipation to an upcoming stressor affects the number of SWDs (Tolmacheva et al., 2012). This might be relevant in case of repeated drug administration studies in which animals get daily injections and the anticipation of this may influence the incidence of SWDs. Once more, a predrug administration EEG recording session will give insight whether there are cross-over effects of a previous treatment or other effects might have happened. A second reason for having a solvent control group is that some solvents, e.g. Tween 80 and a mixture of saline/ethanol/propylene glycol, even when administered systemically, increase the incidence of SWDs (Peeters et al., 1992a). But also i.c. administration of solvents might have an effect on the incidence of SWDs (Tolmacheva and van Luijtelaar, 2007).

In case different doses are used and the relevant doses needs to be explored, it is common to have three different doses, differing a factor 3 from each other. Most often, the effects of a compound vary in time, and dependent on the T max and half-life of a compound the time epochs in which the SWDs are quantified: some drugs have a quick T max and short half-life, and this demands for example 15 min time intervals (as was the case after tiagabine, Coenen et al., 1995) and a post drug recording of 90 min, while other drugs are more slow to be eliminated and the number and duration per hour describes adequately the dynamics of the drug response, this has been the case in for example vigabatrin, some of its effects on incidence lasted 6 h (Bouwman et al., 2007). Therefor it can be expected that other effects of this compound such as a change in spectral power density or mean duration will last more than 48 h. Another example is the experimental anti-absence drug RO0711401, a mGlu1R PAM: it showed a more than 6 h lasting effect on SWD incidence (Ngomba et al., 2011).

Adaptation the animals to the recording leads and cage (for example 24 h) and to the recording room and handling them for a few minutes per day prior to the actual day of the start of the EEG recording session contributes to obtain a reliable and representative EEG measurement. It is our experience that a 2 h base-line recording period prior to the administration of the compound yields a good indication about a subjects hourly number of SWDs and this serves as a solid base to determine changes in the SWD occurrence. Since environmental noise or sudden changes in noise level or ambient light may interrupt ongoing spontaneous behavioral activity, sleep and also SWD, the environment of the recording conditions is critical, without and shielded from environmental noise, and without the presence of equipment and the experimenter in the EEG recording room.

The recording cages should be placed in a light-dark controlled room, similar regarding light on and off as in the vivarium; if not, the animals should be given time to adapt to the shifted LD cycle, it is thought that the speed of adaptation of sleep and SWDs is about 1 h per day (Smyk et al., 2019b).

## Time of Day Effects

The states of vigilance, such as wakefulness, slow wave sleep, or REM sleep, highly influence the occurrence of SWDs. The SWDs preferentially appear during passive wakefulness or during instable vigilance periods such as changes from active wakefulness to passive wakefulness to light slow wave sleep (Lannes et al., 1988; Coenen et al., 1991; Drinkenburg et al., 1991; Smyk et al., 2019a). SWDs rarely occur during deep slow wave sleep, active wakefulness, or REM sleep. Especially deep slow wave sleep, occurring at the beginning of the light period in nocturnal animals, is notorious for its incompatibility with SWD occurrence and during these hours of the 24 L-D cycle rats have indeed few SWDs (van Luijtelaar and Coenen, 1988). The beginning of the light period with its low incidence is an unfavorable period to establish effects of a compound on SWDs. Therefore it is recommended to test the putative anti-absence effects during the dark period, and especially in the first few hours of the dark period the daily maximum in the number of SWDs is reached and the putative anti- or proabsence action can be found more easily than during the light period. Off note, it is imperative to test all animals and all doses at the same time of day in order to control for circadian effects on the occurrence of SWDs, next to the effects of sleep, as well that the compound’s efficacy might depend on the time of day. Circadian rhythmicity on the occurrence of SWDs, albeit difficult to disentangle from the effects of sleep, are well known to be present in absence patients as well as in WAG/Rij rats (van Luijtelaar and Coenen, 1988; Smyk et al., 2011).

### The Technique of EEG Recording

The principles of the EEG recording techniques as used in freely moving animals, reviewed by Coenen et al. (2004), have not changed much, while quite some technical advancements were made. The techniques, as described by Coenen et al. are still state of the art, giving the aims of the recordings, that is establishing whether an acute or chronic administered drug has putative anti- or proabsence effects and on whether the EEG is modified. The latter requires a clean EEG without movement artefacts. However, some technical innovations may open the way for more robust, longer, and more channels recordings.

The nature of the weak electrical signal (microvolts) from the brain demands that it has be amplified before it can be monitored, stored and processed in an adequate way. Commonly, a differential amplification is applied, implying that the differential voltage between the active electrode and ground and between the reference electrode and ground, is used yielding a signal representing the voltage difference between the active and reference electrode (differential recording). The advantage is that the common signals that appear simultaneously and in-phase in both inputs from the active and reference electrodes, often artefacts, are suppressed. This suppression of the common signals is expressed in the “common mode rejection ratio” of the amplifier. Operational amplifiers (OpAmps) are commonly used as EEG amplifiers: they are high-gain electronic voltage amplifiers with an input from an active electrode and one from a reference electrode, both measured against ground, and a single-ended output, which is displayed as the EEG signal between the active and reference electrode. In this configuration, an OpAmp produces an output potential (relative to circuit ground) that is typically hundreds or thousands of times larger than the potential difference between its input terminals. Nowadays OpAmps are available with rail to rail capabilities: this means that they can handle analog signals up to the supply voltages on both the positive and negative rails. A “rail” is a boundary that a signal has to work within, giving it a wide range in amplitudes. Rail to rail implies that OpAmps are able to operate at low supply voltages, relevant in long lasting wireless EEG studies.

Before the EEG signal can be obtained from the OpAmp, a so-called pre-amplifier or front-end amplifier, placed as closely as possible to the signal source, is often used to transfer the small signals from a high resistant circuitry, which is sensitive to all kinds of interfering signals and noise, to a low resistant circuitry, insensitive for disturbing signals. We put the pre-amplifier in the cable plug to the headstage. Also the moving EEG cables, connecting the animals with a swivel, due to grooming, or during an epileptic insult, become relatively insensitive for the induction of interfering signals. Finally, the amplifier should be completely isolated from earth, which guarantees an optimal safety for experimental subjects.

The EEG signal has in principle an infinite bandwidth. Commonly, however, the interest is in a restricted bandwidth in which the relevant phenomena occur. For the EEG this is generally in the range between low frequencies (>0.1–1.0 Hz) and high frequencies (<40–100 Hz). Hence, band-pass filtering is necessary with a high-pass (blocking low frequencies) and a low-pass (blocking high frequencies) filter. Frequencies below 0.1 Hz are difficult to measure in a reliable way, due to gradual changes in properties of recording electrodes or areas around the electrode tips. These slowly changing voltages, called DC-offset or drift, are the reason that by preference an AC (“alternating current”) amplifier is used, rejecting these slow voltages. For a DC (“direct current”) recording (from 0 Hz on) extra measures to reduce offset and drift have to be taken (e.g. by the use of non-polarization electrodes). DC measurements become more and more available and there are indications that seizures, including the generalized SWDs, characterizing absence seizures, are accompanied or preceded by DC shifts.

Depending of the specific experimental goals, adjustment of the high-pass filter from 0.1 to 1.0 Hz is acceptable in most cases, although the rather slow oscillations previously often not recognized or ignored, might have a function in coordinating other electrographic sleep waves such as epochs of delta activity, sleep spindles, and gamma oscillations in recurring groups (Crunelli and Hughes, 2010). The low-pass filter was traditionally set as low as possible, beginning with 30 Hz, over the years more often at 70 Hz or recently higher (200 Hz) to measure also gamma-oscillations (40–80 Hz). Restriction of the bandwidth is done in order to reduce noise, which is not only dependent of circuitry-resistance, but also of bandwidth. The noise of the 50 or 60 Hz electrical net (“hum”) is another complication. Shielding of all cables reduces the problem and the use of a cage of Faraday, an electrically shielded and often sound attenuating chamber in which no electromagnetic radiation can enter, protects the biological signals further from disturbances in the environment. Another solution is a “notch”-filter, specifically designed to block the 50 or 60 Hz hum of the electric network. Nowadays often non-filtered signals are collected; the presence and availability of excelled digital filters allows to filter signals off line, and in some cases on-line. The advantage of recording of non-filtered signals is that apriori no information is removed (=filtered), a disadvantage could be that it is difficult to check the quality of the EEG signals and to monitor a smooth EEG recording session with unfiltered EEG signals. In case high end computers are used, on-line filtering can be performed on the recorded raw EEG and be displayed directly on the monitor. Digital filtering is preferred above analog filtering given that analog filters cause more phase delays, the delays encountered by the digital filtering can be minimal and depend on the quality and speed of the soft-ware.

The unfiltered EEG is sensitive for DC shifts. The amplitude of DC shifts might be up to milliV, and much larger that the amplitude of the EEG (microV). It is our experience, that the direction of DC shift may vary in direction and amplitude across time and space and so it could be different for different electrodes; the DC at the reference electrode could be positive, and simultaneously negative at the active electrodes. Then EEG clipping can easily occur and its accompanying loss of the EEG signals.

The signals from the animal are *via* a connecting cable connected to a swivel, allowing free movement of the animal. Various types of swivels are available, non-motorized and motorized, all with a minimal rotational torque. In case of a large number of channels (> 8), a motorized commutator might be considered. These swivels prevent the connecting cables from tangling and it permits continuous full rotations in any horizontal direction. By adding a spring in the suspension of the EEG cables and an extra loop there are no restrictions toward movements in the vertical direction (rearing). An elegant way to compensate for the weight of the cable in case of mice or young rats are used, is to have a counter weight or counter balance arm allowing the animal to move up and down with its head, lie in different sleeping positions, or stand on its hind legs while still keeping enough tension on the cable to lift or lower the cable, minimizing the cable putting strain onto the animals head, head connector plug or precious headstage.

Finally, the EEG can be recorded and the analog signals have to be digitized. In order to ensure an adequate analysis of the EEG the sampling frequency should be at least twice the highest frequency of interest in the recording (Nyquist’s theorem). The signal is best sampled with a frequency of a power of 2 (2n) (e.g. 256, 512, 1,024 Hz) for a standardized frequency distribution in the spectral analysis. A common sample-frequency at a cut-off of 70 Hz is 256 samples per second. The AD converter should have a resolution of 12 bit. Digitization and data storage are done with a computer using for example a Windaq (Dataq Instruments) acquisition systems, which allows the simultaneous recording and monitoring of multiple channels or animals. Usually there are differences in activity between different brain structures, and the epileptic activity might be generalized to appear only local. Therefore, multiple electrodes may be used to give an adequate reflection of the underlying activity of these different structures.

Due to better electronic component developments, miniaturization, and design of dedicated special purpose components, multichannel-analog amplification, analog-digital converter, encoder, and transmitter might be combined in one single ultra low power component. This allows the design of wireless biosignal transmitters that have adequate size for implantation in small rodents (including mice). The data can be transmitted *via* radiotelemetry and picked up by antenna’s placed around on under the cage, or *via* WiFi or Bluetooth technology *via* a small microcontroller. This microcontroller can already enhance the recorded data by controlling the active ground or reference up to differentiating (compare) local EEG channels up to generating feedback stimuli toward the test animal.

Wireless technology provides the possibility of recording EEG while the animal is engaged in a behavioral test or social interaction test without being hampered by conventional connecting cables, during tonic-clonic seizures without the risk of interfering cables or outside the lab environment (Vyssotski et al., 2006). Wireless recordings prevent mechanical problems as present in hardwired system that makes contact with the free moving animal, and are electrically safe. For a recent example of a multichannel wireless device see Ball et al. (2014).

Instead of the traditional passive electrodes, active electrodes that use active feedback to each electrode can be used. The signal that is sent to active electrodes is the signal from the reference electrode. It can be either a fixed reference electrode, or the mean of a number of active electrodes, called a common mode reference. It is possible to adjust recording settings like sensitivity and or sample rate by sending these adjustments back to the headstage of the animal. Reducing amplification and sample rate can slow down computations in the headstage and lower the amount of data that has to be transmitted. These reductions will result in an enormous reduction of power consumed by the headstage electronics, thus increasing the operational recording time in case wireless technology is used by a longer battery life running time.

Currently the limitations are still the power supply (batteries) of the headstage electronics in wireless systems. Systems are currently under development that can work with a wireless charging of the batteries. However it is unknown whether long time and close exposure to energy-field necessary to charge the batteries could be hazardous for the health and over all wellbeing of the animal. It might also influence the normal natural behavior of the animal (depending on charging performance in relation to magnetic field strength and used charging frequency next to the magnetic field and power of the WiFi- and Bluetooth-frequencies used at close ranges).

Ideally an EEG system would consist of a DC capable measuring multi channel system with high impedance connected to a fast multi channel and high-bit resolution analog to digital converter (with a minimum of 12-bits per channel) recorded by a powerful computer. Data are recorded and stored on hard disk and data are filtered/rectified/analyzed/presented (on screen)/fed back to an active ground/or trigger feedback for subjects evoking a response, such as in case with closed loop deep brain electrical stimulation.

### EEG Electrode Implants

Since the SWDs are generalized and bilaterally symmetrical, registration in a single hemisphere is sufficient, moreover, the exact locations of the cortical EEG recording electrodes do not matter for a large extent after it is clearly established that the oscillations are genuine SWDs, and indeed generalized and bilateral symmetrical, and have the wave form of spike-and waves, as was the case in WAG/Rij rats (Meeren et al., 2002; Sitnikova and van Luijtelaar, 2007). Sleep spindles or some high voltage spindles may appear locally. Many details regarding the surgery can be found elsewhere, regarding the location of the EEG electrodes it can be mentioned that we have excellent experiences with a simple and economical surface electrode implantation and recording system with very few cable movement artefacts. First, we implant three epidural electrodes, most common one is implanted above the frontal cortex, (2 mm anterior to bregma and 2 mm lateral). This location above the frontal cortex is excellent for recording delta waves, which are more clear expressed at the frontal cortex than at the parietal cortex, as well as for recording the most clear SWDs as the amplitude of the spikes of the SWDs in WAG/Rij rats is most pronounced expressed at the frontal cortex (Midzianovskaia et al., 2001; Meeren et al., 2002). A second electrode is preferential at the parietal cortex, ca 4 mm lateral and 6 mm posterior to bregma. It serves as a second active electrode or as a reference and it records theta activity originating from the hippocampus beneath the cortex. This might be necessary in case sleep and REM sleep need to be analyzed. For a clean EEG, a third, earth, electrode is helpful, it enables a good electrical potential measurement of all other (active and reference) electrodes. The usage of an earth electrode enhances also the common mode suppression and in combination with an active and reference it gives a way more stable signal. It is connected to the ground of the amplifier. We have good experiences pacing this electrode above the cerebellum. Plastic One Inc., Roanoke, USA, tripolar electrode sets MS 333/2 were often used by us. Alternatively, different types of pre-fabricated or in-house made electrodes can be used, for example for recording more than two to six cortical areas (see Meeren et al. (2002) from an epidural grid, from a set of (24) screws in the cranium (Ding et al., 2019), from a set of pre-prepared electrodes glued in a pedestal for cortical and subcortical recording (Lüttjohann and van Luijtelaar, 2015), or with Neurnexus probes or macroarrays (Jonak et al., 2018), or with self-designed alternatives allowing 24 EEG channel recordings in free moving mice (Wasilczuk et al., 2016; Ding et al., 2019). These can be custom made regarding locations and depth and prefabricated. Electrodes can be constructed from fine stainless steel wire, connected to a pin with a small insert. A large number of electrodes that are individually wired and connected with an external recording system quickly demands space on the animals head and this may be challenging. A solution can be found by the usage of multiplexed headstages: they overcome this limitation by combining the signals from many electrodes to a smaller number of connections directly on the animal’s head (Wang et al., 2017).

Once the EEG electrode set is implanted, and the animals are given a recovery period of 1 to 2 weeks, the animals are handled for a few days and habituated to the recording conditions by connecting them *via* a swivel. Next, the EEG can be recorded in freely moving animals for several hours or days. In order to see whether the EEG signals are of a sufficient quality and have a biological origin (instead of filtered noise), it might be necessary to see whether the amplitude of the EEG signal increases when the rats are passive and asleep, or when behaviorally active, theta activity might be present, dependent on whether the electrode is placed in or above the large hippocampal area in rats.

For methodologies that require a complete immobility of the animals [e.g., *in vivo* extra and intracellular electrophysiology, magnetic resonance imaging (fMRI)], most anesthetic drugs cannot be used as they suppress SWD. Instead, neurolept-analgesia (Hypnorm, a mix of fentanyl and fluanisone, see Inoue et al. (1994) for details and different doses), and local injections of lidocaine have been used to allow the contention of the animals into a stereotaxic frame (Pinault et al., 1998). Using this preparation, spontaneous SWD and extracellular unit activity can be recorded with the same pattern and occurrence as in the awake freely moving rats (Pinault et al., 2001; Deransart et al., 2003), although an increase in number of SWD and a decrease in the interspike frequency has been found in WAG/Rij rats after anesthetic doses of Hypnorm (Inoue et al., 1994). Moreover, a putative new anti-absence drug should be investigated in freely moving animals while the behavior being quantified.

### The Dependent Variables

**Figure 2:**
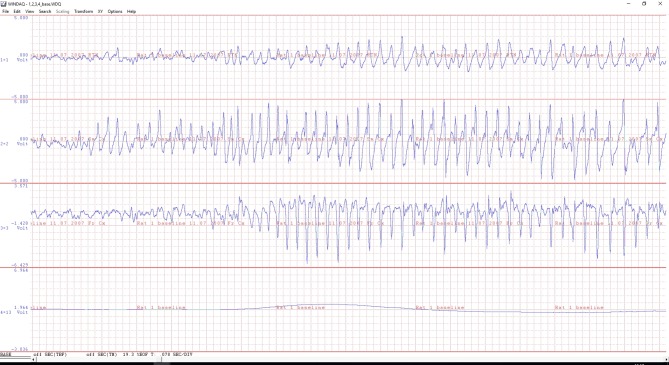
An example of a ECoG of a WAG/Rij rat with electrodes in the reticular thalamic nucleus (upper channel), sensory motor (middle) and frontal (bottom ECoG) cortex. The beginning of a 8 Hz spike-wave discharge (SWD) is illustrated. Note the differences in morphology between the three EEG traces during the generalized SWD. The fourth signal is from an infrared movement detector (PIR); the line is flat when the animal is motionless, while showing large and steep changes during active movements (not illustrated). A minor body movement is seen at the beginning of the SWDs. The time scale of the X-axis is 0.078 sec/division. The amplitude, voltage, is on an arbitrary scale for monitoring purposes only.

The EEG’s are analyzed regarding the presence of SWDs. An example of a SWD, as measured in cortex and thalamus, is depicted in **Figure 2**. The parameters that are analyzed and reported are the number of SWDs (incidence), mean duration of SWDs, SWD-index, total time of SWD activity, and all per time unit, are most commonly used to quantify SWDs. SWD index and total time of SWD activity are less favorable since both measures are a composite of the number/incidence and mean duration. Moreover, the number of SWDs is mainly determined by the excitability of the cortex, the mean duration is determined by different endogenous mechanisms involved in aborting ongoing SWDs in which the reticular thalamic nucleus plays a major role (Lüttjohann and van Luijtelaar, 2015). By reporting the composite parameter, one loses the occasion to report on the different processes controlling SWDs. Some drugs might have specific effects on the number of SWDs; other drugs might influence their mean duration. The hazard rate of the durations of the inter-SWD intervals has been used by e.g. Bouwman et al. (2007): it indicates the instantaneous probability that the SWD will stop as a function of time and reveals information about the underlying SWD stopping mechanism (Maris et al., 2006). It was changed after the anticonvulsant and proabsence drug vigabatrin, leading to an enhancement of rather short SWDs as well as an increase in the number of rather long SWDs.

The scoring of the SWDs is most often done by visual inspection, which is still the golden standard. For criteria and see van Luijtelaar and Coenen, 1986; and the next paragraph for a discussion. Automated scoring systems for SWD quantification in WAG/Rij rats were developed by us (Westerhuis et al., 1996 and adapted to SpikeWave Complex Finder software by PLC van den Broek, Radboud University, Nijmegen). This and other automated system should be considered as auxiliary tools for the analyses of large amounts of data. Considering the rather stereotypical appearance of SWDs it is not difficult to develop a sensitive, selective, and reliable SWD quantification system (for review see van Luijtelaar et al., 2016; Casillas-Espinosa et al., 2019).

In all cases it is recommended to observe the rat’s behavior after the administration of an unknown drug since the animal’s behavior may changes qualitatively (signs of psychogenic behavior), and stereotyped behavior and clonic seizures might show the same quantitative activation pattern as grooming. This difference cannot always be appreciated by fully automated movement detection or registration systems. The possibility exists that a putative antiabsence drug or an experimental drug may reduce SWDs because the compound changes the animal’s behavior. An illustrative example is cocaine; nobody would claim that it is a good anti-epileptic or anti-absence drug although it reduced dose-dependently SWDs in WAG/Rij rats. Cocaine eliminated explorative and automatic, and passive behavior, whereas various stereotypical activities such as uncoordinated head and body movements and head swaying emerged. In total bodily activity was increased by cocaine, and similar observations can be seen after the administration of amphetamine. This was detected by systematic behavioral observations and quantification of the observed behavior (van Luijtelaar et al., 1996). In these cases the reduction of SWDs is secondary to the increase in physical/bodily activity of the animal induced by the drug. Another example would be the central stimulant apomorphine. It does reduce the number of SWDs (Midzianovskaia et al., 2001), but this decrease is secondary to the effects of apomorphine on behavior.

In case an increase in passive behavior is found, than it might be worthwhile to analyze whether the drug is affecting sleep quality and quantity. If it does, than the benefits of good seizure control should outweigh the negative effects on sleep quality and or quantity. Therefore, it is imperative to get an impression whether the drug has sedative effects or activating effects on behavior. Although this information can be to some extend be obtained from the EEG recordings, REM sleep can be determined in rats only if together with the EEG and electroomyogram (EMG) or an index of the animals behavior (active, such as walking, explorative behavior, grooming, eating, drinking, or being passive) or passive (lying, sitting with eyes open or closed) is available. A simple, reliable, and cheap alternative to determine changes in behavior (either a decrease or increase) of a compound is to have an infrared movement detector (PIR, Passive Infrared Recorder) above each recording cage. In case a new compound has sedative or hypnotic effects, the PIR will register less bodily activity. In that case it is recommended to analyze the EEG together with the PIR in terms of sleep parameters. Sleep can be either qualitatively of quantitatively affected. Quantitative aspects are e.g. described by percentages total sleep, non-REM sleep, or REM sleep and by the number and duration of intermittent wakefulness periods, or sleep efficacy, for a study of the effects of the anti-absence drug ETX on sleep see van Luijtelaar et al. (2012b). A major qualitative aspect of non-REM sleep is the amount of delta activity, by visual inspection of the sleep EEG it is difficult to establish the amount of specifically deep slow wave sleep. Spectral analyses of non-REM sleep is then indicated with a focus on the amplitude of delta activity (in rats 1–5 Hz). Amplitude or power can be expressed as percentage of total power over 0.5–100 Hz to normalize the data across animals. Delta power can be increased or decreased compared to control treatment, benzodiazepines, or its partial agonists are known to reduce delta power (Coenen et al., 1992), the pro-absence drug tiagabine facilitates delta power (Lancel et al., 1998). The nuchal EMG is most often used for visual and automatic sleep wake classification in rats, a good replacement for the nuchal EMG is the signal obtained from a PIR. As mentioned, it monitors the amount of bodily activity of an animal in a recording cage; the amplitude of the analog signal from the PIR is high when the animals are physically active, and the amplitude is low when the animal is passive. The analyses of the amplitude of this signal (the area under the curve) from a PIR will yield the first indication whether the behavior of the animals is changed as a consequence of the administration of the drug.

### Are All 8 Hz Oscillations SWDs?

A relevant question that was recently raised again is whether all oscillations in the 8 Hz domain, mimicking to some extent the SWDs in GAERS and WAG/Rij rats and are known for a long term to be present in different outbred strains of rats (Willoughby and Mackenzie, 1992; Marescaux et al., 1984; van Luijtelaar et al., 1994) and now noticed in wild rats, should be indeed considered as genuine epileptic SWDs (Taylor et al., 2017; Taylor et al., 2019). First, WAG/Rij rats have clinical concomitants during the SWDs, they are mild, and might be easily overlooked. A combined EEG video study showed that during the cortical expressed SWDs WAG/Rij rats show head tilting, twitching of the vibrissae, accelerated breathing, and occasional eye blinks, and facial myoclonus in otherwise immobile animals (van Luijtelaar and Coenen, 1986). Close inspecting of the EEG oscillations as presented in Taylor et al. (2019) shows that only a minority of the illustrated SWDs in the wild rats could classify as SWDs according to our criteria and characteristic features. Relevant and typical is that SWDs appear as bilateral symmetrical generalized, minimal duration 1 s, asymmetric morphology with a sharp large amplitude negative peak with a duration between 25 and 35 ms, this peak is most pronounced expressed at the frontal cortex and a clear negative wave (40–60 ms), less well expressed at the frontal cortex but more at the thalamic VPM, with a “sudden” appearance from a normal appearing background and without a waxing and waning pattern). The possibility that the 8 Hz oscillations as seen in the wild rats represent a local sensory motor rhythm, often described in cats (it was also called mu-rhythm) but in some rodents strains as well, characteristic for attentive wakefulness, remains to be explored. Although we have never seen a clear sensory motor rhythm in WAG/Rij rats, it is likely to be present in other outbred lines such as Long Evans rats. In GAERS, the SWDs seem to be driven by a 5–9 Hz oscillation, as was originally proposed by Pinault (2003). Moreover, WAG/Rij do not have SWDs while being attentive or aroused (Osterhagen et al., 2010).

We did describe (Drinkenburg et al., 1993) also 8 Hz SWD-like phenomena in the cortical EEG of WAG/Rij rats during drowsiness and light slow wave sleep, not fully meeting our rather strict criteria of SWDs and named them spiky phenomena; their appearance is in between sleep spindles and SWDs, less sharp spikes, more symmetric than SWDs, poorly expressed wave, and a waxing and waning pattern, a shorter duration than SWDs, rarely exceeding 2–3 s, mimicking also some of the high voltage spindles, as were introduced by Buzsáki and were recorded during alert immobility (Buzsáki et al., 1988; Jandó et al., 1995). The fact that quite some WAG/Rij rats have spiky phenomena next to genuine SWDs, demonstrates that there is a continuum regarding the morphology of the 8 Hz oscillations. Moreover, sometimes a genuine appearing SWDs transfers into a spiky-phenomenon. The spiky phenomena are less well studied, and it is not known whether they are indeed generalized, are bilateral symmetrical have a similar cortical origin as SWDs, and whether they are accompanied by clinical concomitants. The fact that the “peaks” of the spiky phenomena are less sharp than in genuine SWDs casts serious doubts about them being epileptic (an epileptic spike is per definition sharp). The sharpness of the peak of the SWDs is mostly clearly revealed by the presence of the second and higher harmonics of the dominant frequency spectrum of SWDs, their amplitude, or power is lower in spiky phenomena and sleep spindles compared to SWDs. In fact the energy in the 30–80 Hz band (Ovchinnikov et al., 2010) and the slope of the frontal peaks of the SWDs (SWD detection and quantification program developed by P. van den Broek, and used by us for more than 15 years and by the group of Terence O’Brien, Melbourne) were empirically found as the most sensitive and reliable parameters for the automatic detection of SWDs and to separate SWDs from other 8 Hz oscillations, among others the intermediate sleep spindles characterizing the transition between nonREM and REM sleep (Gottesmann and Gandolfo, 1986). But there are many other methods to detect SWDs in rodents, for review see van Luijtelaar et al. (2016). Another way to distinguish epileptic SWDs from non-epileptic 8 Hz oscillations is the presence of a second small negative spike in a SWD, just before the large amplitude negative spike dominating SWDs. It appeared in SWDs at the occipital cortex and in VPM, this small spike survived an SWD averaging process, suggesting that it is genuine (Sitnikova and van Luijtelaar, 2007). This small spike characterized SWDs in 44% of the patients, as was described in a classical study by Weir (1965). Finally, a single Morlet based model could reliable classify SWDs from other 8 Hz oscillations (Sitnikova et al., 2009), demonstrating a different morphology than the other 8-Hz oscillations as can be found in the cortical EEG of WAG/Rij rats. Besides the morphological differences between SWDs and other 8 Hz oscillations, there is the predictive validity based on the evaluation of at least 12 different anti-epileptic drugs of the models, there is little doubt about that the SWDs seen in the WAG/Rij and GAERS models are truly epileptic.

### Some New Principles for EEG Data Analyses

The EEG was used classically for basic research and clinical applications on the field of epilepsy and sleep; and tended to become less relevant for basic neuroscience research through the availability of functional MRI and other brain imaging techniques, an analyses of PubMed showed that over the years the number of rodent EEG papers is increasing again. The classical way to analyze the EEG in case of clinical applications in the field of epilepsy is still visual inspection (“the golden standard”), mainly regarding the description of aberrant EEG activity regarding morphology and spatial temporal distribution, and frequency and duration of occurring of interictal spikes, or other epileptoformic electroencephalographic characteristics, including the SWDs. The availability of computers and software for single channel EEG analyses allows to describe the spectral content of the EEG and the effects of drugs, including the effects of antiepileptic drugs on the spectral content of the EEG drugs. Of later development are the spectral decomposition techniques allowing to describe its changes from moment to moment, e.g. by wavelet analyses. These spectral analytical techniques have revealed the dynamics within SWDs (Bosnyakova et al., 2007), but also differences between preictal, ictal, and interictal EEG activity. The presence of multichannel EEG, at least more than one single channel, allows a new category of analyses, network analyses. It allows evaluating neuronal synchrony in EEG data, either obtained with invasive as the case with epidural or depth electrodes commonly used in rodent EEG based sleep and epilepsy research, or non-invasive recording techniques in human EEG or MEG studies. Different methods can be used, for a tutorial review see Bastos and Schoffelen (2016), such as coherence, a robust measure which is commonly used as an index of the non-directed functional coupling between different brain structures at various frequencies (Thatcher, 2012), linear and non-linear correlation function (Lopes da Silva et al., 1989), phase synchronization index, mutual information function, transfer entropy, and partial directed coherence, and Granger causality. It allows to describe, in the time domain, but sometimes as well in the frequency domain, the relation between the activity in two or more EEG channels, as well, depending on the method, the directed functional relationships, in the sense that it can be established which channel influences the other channel(s) and at which strength, and its dynamics in case a sliding window is used. We have successfully applied some of network analyses techniques to describe dynamics in coupling and based on a non-linear regression technique a cortical onset zone was described in the WAG/Rij rats, which was the bases for the cortical focus theory (Meeren et al., 2002; Meeren et al., 2005; Westmijse et al., 2009; van Luijtelaar et al., 2011). Next, different thalamic nuclei have a different function in the maintenance and abortion process of SWDs, as was established with different network analyses techniques (Lüttjohann and van Luijtelaar, 2015; Sysoeva et al., 2016). Drug related studies with network analyses methods of antiepileptic drugs are scares, in other domains they were already successfully applied (Ahnaou et al., 2014) and there are good reasons to assume that new mechanisms and new drug targets can be found with these new approaches acknowledging that epilepsy and certainly genetic generalized epilepsy, are network types of brain dysfunctions (Stefan and Lopes da Silva, 2013; Lüttjohann and van Luijtelaar, 2015).

### Limitations and Strength.

A limitation of the WAG/Rij model is that this model, the same holds for all other genetic absence models, does not show the spontaneous remission (different studies show a range from 21%–74%), or change to generalized convulsive seizures occurring in ca 40% of children with childhood absence epilepsy (Tenney and Glauser, 2013). Another limitation is that it has a single genotype, and the heterogeneousness regarding genetic causes, commonly found in children, is missing in this inbred strain. The strength is that the genetic models are well characterized, have face, predictive, and construct validity, that the rats (WAG/Rij) are widely available (also commercially at Charles River), that different groups report the same epileptic phenotype. Epigenetic effects are described, and comorbidities, present in epilepsy patients such as dysthymia, are modeled as well (Sarkisiva et al., 2010). Finally, the WAG/Rij and GAERS models have no other obvious neurological deficits and allow the study and the development of seizures in a genuine epileptic brain.

## Author Contributions

GvL and GvO compiled and wrote the manuscript.

## Conflict of Interest

The authors declare that the research was conducted in the absence of any commercial or financial relationships that could be construed as a potential conflict of interest.
